# Musculoskeletal education: a curriculum evaluation at one university

**DOI:** 10.1186/1472-6920-10-93

**Published:** 2010-12-12

**Authors:** Marcia L Clark, Carol R Hutchison, Jocelyn M Lockyer

**Affiliations:** 1 Department of Surgery, Faculty of Medicine and Dentistry, University of Alberta Edmonton, Alberta Canada; 2 Faculty of Medicine, University of Calgary, Calgary, Alberta Canada; 3 Department of Community Health Sciences, Faculty of Medicine, University of Calgary, Calgary, Alberta Canada

## Abstract

**Background:**

The increasing burden of illness related to musculoskeletal diseases makes it essential that attention be paid to musculoskeletal education in medical schools. This case study examines the undergraduate musculoskeletal curriculum at one medical school.

**Methods:**

A case study research methodology used quantitative and qualitative approaches to systematically examine the undergraduate musculoskeletal course at the University of Calgary (Alberta, Canada) Faculty of Medicine. The aim of the study was to understand the strengths and weaknesses of the curriculum guided by four questions: (1) Was the course structured according to standard principles for curriculum design as described in the Kern framework? (2) How did students and faculty perceive the course? (3) Was the assessment of the students valid and reliable? (4) Were the course evaluations completed by student and faculty valid and reliable?

****Results**:**

The analysis showed that the structure of the musculoskeletal course mapped to many components of Kern's framework in course design. The course had a high level of commitment by teachers, included a valid and reliable final examination, and valid evaluation questionnaires that provided relevant information to assess curriculum function. The curricular review identified several weaknesses in the course: the apparent absence of a formalized needs assessment, course objectives that were not specific or measurable, poor development of clinical presentations, small group sessions that exceeded normal 'small group' sizes, and poor alignment between the course objectives, examination blueprint and the examination. Both students and faculty members perceived the same strengths and weaknesses in the curriculum. Course evaluation data provided information that was consistent with the findings from the interviews with the key stakeholders.

**Conclusions:**

The case study approach using the Kern framework and selected questions provided a robust way to assess a curriculum, identify its strengths and weaknesses and guide improvements.

## Background

Musculoskeletal education has been recognized as a national and global priority[1-6] during the Bone and Joint Decade (2000-2010). In both the United States and Canada, it was recognized that musculoskeletal education and the treatment of musculoskeletal diseases have had insufficient attention in medical school curricula[1,2,5]. In response to these findings, the Association of American Medical Colleges (AAMC) undertook a review to identify learning objectives on musculoskeletal conditions and suggested ways to better target these objectives in medical school curricula[2].

The University of Calgary, Faculty of Medicine's three year curriculum is based on the Clinical Presentation model. These Clinical Presentations encompass the 120 ways that patients present to a physician[3]. In the overall undergraduate curriculum, algorithms for learning clinical presentations and clinical schemes[7] are used to approach, organize and synthesize clinical problems[7,8]. This approach has demonstrated its ability to allow students to organize their knowledge and develop their problem-solving skills[7].

**Figure 1:**
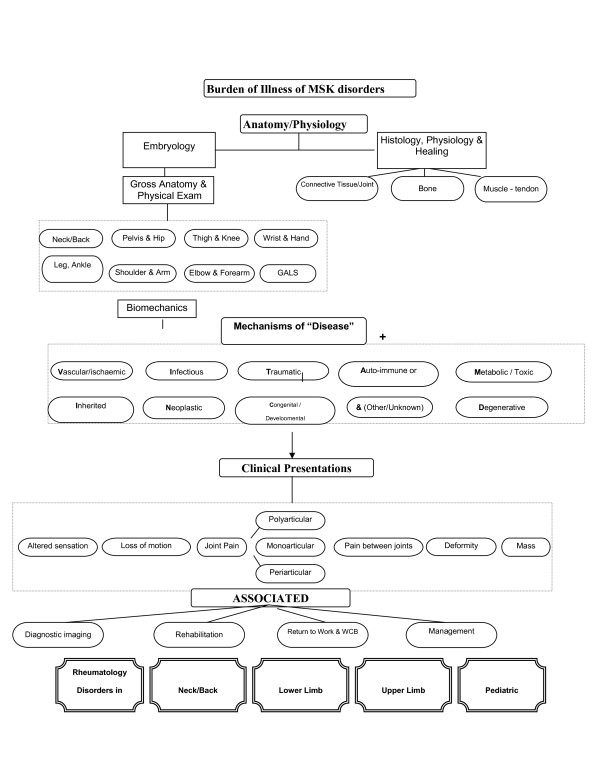
**Burden of illness of MSK disorders**.

**Figure 2:**
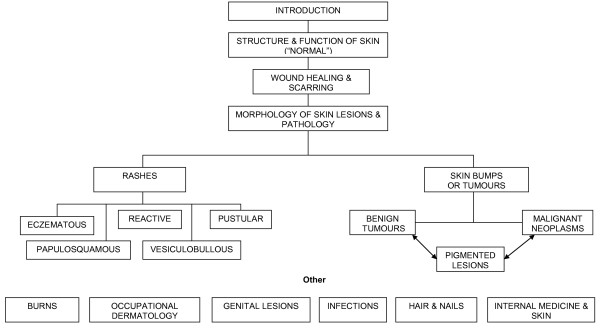
**Skin Concept Map**.

The musculoskeletal (MSK) course was the second of the seven courses. The course comprised three main sections. These were dermatology, musculoskeletal medicine and special senses. Musculoskeletal medicine (MSK) included the topics and specialties of anatomy (histology, embryology, gross and clinical examination), rheumatology and orthopaedic medicine. Special senses included the topics relating to balance, vision, hearing, voice and sight or components of specialties of neurology and ear/nose and throat (ENT) medicine. The course followed the overarching themes of Clinical Presentations (Table 1) and concept maps (Figure 1 and 2). There was 125 hours of formal instruction with 85 hours of lecture time and 31 hours of small group time scheduled.

**Table 1:** Clinical Presentations for MSK Course

Painful Limb
• Painful Swollen Limb
• Venous Thrombosis and Hyper-coagulable state
• Intermittent claudication

Hair and Nail Complaints

Skin Tumors, Benign and Malignant

Skin Blisters

Skin Rash (Dermatitis)

Joint Pain, Mono-Articular (Acute, Chronic)

Joint Pain, Polyarticular (Acute, Chronic)

Regional Pain, Non-Articular (Hand, Wrist, Elbow, Shoulder, Spine, Hips, Knee, Foot)

Skin Lesions and Systemic Disease
• Skin/Immunologic Diseases
• Primary and Secondary Lesions (Structure & Function of Skin)

Fractures and Dislocations

Ear Pain Hearing Loss, and Tinnitus

Vision Loss
• Chronic Visual Loss
• Acute Vision Loss

Eye Redness
• Red Eye
• Eye Injuries

Student feedback from the first class who participated in the revised curriculum showed that the MSK course had performed sub-optimally. The new course did not appear to address the weaknesses that the MSK curriculum had consistently identified over the previous 10 years. It was agreed by senior faculty that an in-depth examination of the curriculum was warranted so improvements could be made to the course before its next iteration. In addition, the Faculty of Medicine was scheduled for an accreditation survey in the coming academic year, which provided a major opportunity for this course and others to be reviewed. The purpose of this study was to use a case study approach to examine the strengths and weaknesses of the MSK course. This study took place from 2007 to 2008 looking at data from the Class of 2009 (i.e. the cohort who began in September 2006). The course occurred in the fall of 2006.

## Methods

The authors selected a mixed-methods case study approach for this study in order to draw upon both quantitative and qualitative approaches to systematically examine the curriculum[9]. A case study allows examination of a complex system from a number of perspectives within a real-life context[9-11]. For the purpose of this study, the authors examined multiple data sources including course documents, the course's multiple choice question (MCQ) examination, and summary data from student and teacher course evaluation data (Table 2). The authors also conducted semi-structured interviews with students and key faculty members.

**Table 2:** Course Documents Accessed for Case Study Research Findings

Accreditation documents and website http://www.ucalgary.ca/medaccreditation/node/30/
Strategic Planning documents

Liaison Committee for Medical Education website information http://www.lcme.org

Personal Communication with the Associate Dean of UGME

End of Course Evaluation - Students

End of Course Evaluation - Faculty

Course Chair Report

Mean total scores, end of course evaluations Class of 2005-2009

Interview data from key informants

Class of 2009 Student Handbook

Student Evaluation Policy

Core Syllabus

Undergraduate Medical Education Clinical Correlation Information and Responsibilities for Preceptors and Students.

Concept map - Skin and MSK

Curriculum Information System - student resource base

Computer Disks given to students - Physical Exam Skills of the MSK system and Approach to Rheumatologic Diseases

Handbook of procedural skills

Personal Communications with students and administrative personnel.

Faculty Listing for Teaching of MSK course

Course Curriculum Committee Minutes

Student Evaluation Policy

Course Blueprints

Summative Examination - Outline

This case study aimed to understand the strengths and weaknesses of the MSK curriculum. It was guided by four questions. (1) Was the course structured according to standard principles for curricular design? (2) How did students and faculty perceive the course? (3) Was the assessment of the students (i.e., through a multiple choice examination) valid and reliable? (4) Were student and faculty course evaluations valid and reliable?

**Figure 3:**
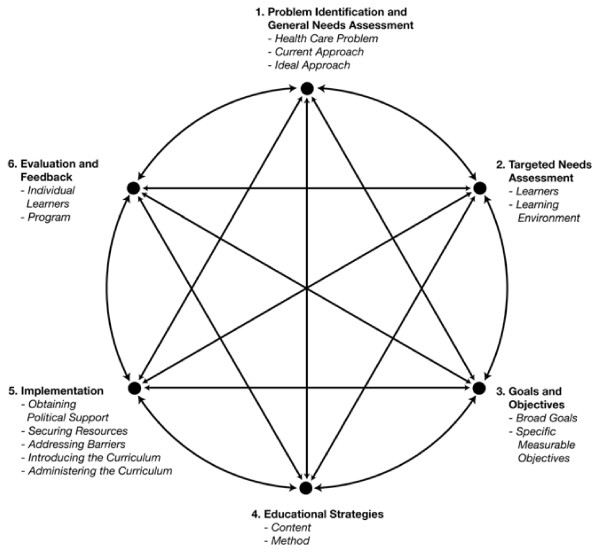
**Kern, David E., M.PH., Patricia A. Thomas, M.D., Donna M. Howard, R.N., Dr.P.H., and Eric B. Bass**. Curriculum Development for Medical Education: A Six-Step Approach. p.6, Figure 1.1. ^© ^1998, 2009 The Johns Hopkins University Press. Reprinted with permission of The Johns Hopkins University Press.

To address the first question related to course design, we drew on Kern, Thomas and Hughes (Kern)[12] curriculum evaluation framework (Figure 3) in which a six step, iterative process of evaluation and feedback guides curriculum development and assessment. According to the Kern model[12], the various components of a well designed and delivered course should be aligned and congruent with one another. As part of the Kern model[12], changes in a course's objectives will inform the curriculum and the approach to evaluating students. Similarly, if new content is introduced, objectives will need to be re-examined and the evaluation of the curriculum adjusted.

To this end, course documents were examined for evidence that the MSK curriculum aligned to the Kern framework[12]. The authors looked at documents to determine: (1) what problem(s) or needs guided the initiation of the curriculum; (2) was a needs assessment undertaken to determine learners needs; (3) were there measurable and specific goals to guide the course; (4) did the course objectives encompassed cognitive, affective, psychomotor, process and outcome domains[12]; (5) were a variety of learning strategies that matched the objectives being used; (6) were course resources (such as personnel, funding and facilities) suitable and was there internal and external support for the curriculum; and (7) were students, teachers and the curriculum evaluated in a valid and reliable manner.

To determine the perceptions of students and faculty about the strengths and weaknesses of the course, the authors conducted interviews specifically asking about the course's strengths and weaknesses, areas believed to require improvement and areas that included inadequate content. The authors analyzed this data along with the open-ended comments provided by students and faculty on the evaluation forms. Each interview was conducted in person by the principle investigator and recorded for transcription and analysis. Interviews were transcribed verbatim taken during the interview were recorded and included in the interview data. The analysis of interviews began with the first interview where themes and sub-themes were identified and coded. Using a constant comparison technique, each subsequent interview was coded and new themes/sub-themes were added. The coding from previous interviews was adjusted as needed[13,14]. Thus analysis occurred in an iterative manner to ensure that new themes encountered included data from prior interviews. The process of adding and modifying themes and interviewing continued until no new themes emerged. Congruence and validation of the coding structure and thematic analysis were triangulated through discussion with other research team members. The researchers all reviewed the transcripts and coding structure after the second and fourth interviews. After 10 interviews had been conducted, no new themes were identified, thus data was considered to be 'saturated' and no further interviews were conducted.

The open ended comments on the course evaluation survey for both faculty and students were analysed through thematic coding with attention to generated themes. Each comment, organized by subject heading, was read and analysed in an iterative manner with a thematic coding structure created. If new themes emerged, the coding structure was revised and the previous comments read again to determine congruence with the new themes. Further comments were read with the coding in mind and additional codes or themes were added if they emerged.

Recognizing that it is difficult to draw meaningful inferences about student performance without valid and reliable data, the authors assessed the MSK course summative examination for evidence of validity and reliability. First, the data from the 90 multiple choice questions (MCQ - "one-best-answer" with four choices) were analyzed using Cronbach's alpha to assess for internal consistency reliability. The researchers assessed validity qualitatively by examining the core syllabus, exam blueprint, MCQ examination and course schedule to establish the proportion of curricular content tested on the examination. Each of the 90 items on the examination was assessed to determine whether the knowledge being assessed mapped to the course blueprint. Lastly, the authors reviewed objectives in the core syllabus to determine whether there was congruence and proportionality between the objectives and the examination.

To assess the quality of the student and faculty course surveys (i.e. feedback on the course), the authors examined these data for evidence of reliability and validity. The student evaluation consisted of 52 items on a five point scale. Data from the student evaluations for the previous two years were also collected as the three data sets contained 24 items that were continuous over the six years. The faculty feedback dataset consisted of 14 questions in the form of a five point scale. Faculty data from two previous years were collected and used in analysis as well.

Both data sets were analysed for descriptive statistics, a detailed examination of the quality of the items[15] and calculation of internal consistency reliability (Cronbach's alpha). Additionally, a one way analysis of variance (ANOVA) was performed to determine whether a statistically significant difference existed between the total mean survey ratings to establish construct validity (comparison of means from three different iterations). Validity for both surveys was determined through comparison to Berk's criteria[16].

As a final step in determining the strengths and weaknesses of the curriculum, the researchers triangulated the data from all of the sources. Triangulation is the process of "corroborating evidence from different individuals, types of data and methods of data (i.e. documents and interviews) to support a theme"[13].

Ethics for this study was obtained by the University of Calgary, Office of Medical Bioethics.

## Results

In the fall of 2006, the course was delivered to 139 students, over 31 formal instructional days. The medical students were selected for medical school, based on their Grade Point Average (after completing at least two years of full time university), Medical College Admission Test(MCAT), Essays, and an Interview Process as outlined in Brownell et al [17]. Teaching for the course was delivered through a mix of didactic and experiential learning methods. Students received a total of 85 hours of lecture time and 31 hours of small group time (125 hours of formal instruction) from 101 teachers (full-time and clinical faculty as well as residents and fellows). The stated number of faculty (101) were needed to assist with lectures and the small group format.

For the purposes of clarity, the word faculty is used to include faculty members, residents, and Fellows: Residents are physicians who have received their M.D., and are obtaining additional training in specialty areas of medicine such as internal medicine or surgery. Fellows have passed all of their licensing examinations and they are obtaining further sub-specialty training before beginning their practice. Faculty were practicing physicians in the specialties represented by the course. The majority had private practices outside the University setting. Due to the retrospective nature of the study, detailed information about the pedagogic, scientific and clinical experience as well as formal education of the 101 faculty used to deliver the course was not obtainable.

1) Curricular review as outlined by the Kern model[12].

The Kern framework[12] was used to determine whether a needs assessment at the University of Calgary had been guided by recommendations from international organizations (United States Bone and Joint Decade[1]) in the development of the MSK curriculum. Previous course evaluations at the Faculty of Medicine had identified that the MSK course needed improvement. The objectives for the curriculum, as found in the core syllabus, had deficiencies with respect to format, organization and specific/measurable content. The layout, language and organization of the course objectives were inconsistent and difficult to comprehend. Also, the objectives were not consistently presented in a specific or measurable manner as recommended by Kern[12]. The course used a number of educational strategies, as defined in the Kern model, to deliver the content of the curriculum including lectures, problem solving, discussion, group learning, demonstration of real life experiences, digital learning aids, prescribed reading and standardized patient experiences.

Implementation of the MSK course required a large number of teachers and significant resources to achieve the active learning requirement that had been set for the general medical school curriculum. Faculty resources (number of faculty, time) were stretched. For example "small" group sessions were large (> 14 students), which did not align with the Faculty's own guideline for small group size of <14 students, as documented in curricular handbooks[18].

Both faculty and students identified that insufficient time was available to master the content particularly because there was a lack of integration of content and flow of new material. This was supported through analysis of curricular documents and key informant information. It was also communicated through a reported sense of disorganization of the course in the interview data. The lack of integration of content and disorganization also supported the comments that there was not enough time to deliver or comprehend the course content. Both the students and faculty felt that more time was needed to present the content of the course, as represented by the results the interviews and survey data.

The course was evaluated by both students and faculty; but there was no mechanism for faculty to receive feedback about their performance, a key aspect of the Kern model[12].

2) Course perceptions.

Interview and open ended survey data showed that both faculty and students identified the commitment and clinical experience of teaching faculty as course strengths. This data set also identified a number of weaknesses in the course: the large number of faculty needed to deliver the course; the large size of small group learning activities; the timing of small group sessions; time constraints on course content delivery; and the addition of special senses to the curriculum. Data suggested that improvements were needed in organization/scheduling of the curriculum, provision of additional small group learning opportunities and using clinical schemes[7] for teaching. A key finding was the need to improve the core syllabus by establishing objectives and devising a strategy to link the objectives with the assessment. Interviewees also suggested having a core group of teachers with training to improve teaching delivery methods. The data also supported developing a method for faculty to review student feedback with regards to their teaching performance.

3) Reliability and Validity Assessment of the exam.

The student multiple choice question (MCQ) exam was assessed for evidence of reliability and validity. The Cronbach's alpha for internal consistency reliability was r = 0.76, p < 0.05 suggesting a reasonable reliability. The examination of content validity considered the alignment of course objectives with the examination blueprint and the multiple questions. It indicated that the MCQ was not well aligned with the objectives or the examination blueprint provided to the students. Nonetheless, the course MCQ appeared to have concurrent validity as the Pearson r correlation between the MSK course and the preceding course and the MSK and the next course were r = 0.68, p = 0.01 and r = 0.63, p = 0.01, respectively. These data suggest a positive correlation in scores between examinations and that students who did well in this course did well in other courses.

4) Reliability and validity of the course feedback.

Both the student and faculty evaluation forms for the course demonstrated reliability (r = 0.92 student, r = 0.76 faculty, p < 0.05). When the results of the evaluation for the current iteration were compared with previous years, there was a statistically significant difference found, suggesting the current course performed less well (Df 2,218 F = 10.77, p = .000). The faculty did not rate the course differently compared to previous iterations. Validity assessment of both the student and faculty survey aligned with Berk's criteria[16] but did identify a few inappropriately worded items (e.g. items that contained more than one discrete idea).

## Strengths and weaknesses of the course

The course had several strengths as summarized in Tables 3, 4 and 5; as evident from the analysis using the Kern framework[12]. These strengths included a curriculum in which most of the components of the Kern framework[12] were adhered to when the course was designed. Specifically, there was a diversity of educational strategies designed to maximize the opportunities for knowledge, skills and attitudes to be developed, good implementation of the curriculum with a high level of commitment to teaching evident from both learner and teacher perspectives, a reliable examination to assess student learning, and evaluation questionnaires for the course that provided relevant information to assess the function of the curriculum and guide its future development.

**Table 3:** Strengths and Weaknesses of the MSK Curriculum

Was MSK course structured according to the standard principles for curriculum design?				
**Kern Step**	**Data sources**	**Strengths**	**Weaknesses**	**Exemplar Quotes**

Problem identification and general needs assessment conducted	Data identified in Table 1	Course evaluation feedback from students and faculty used to revise curriculum.	No evidence that demographic, patient, hospitalization or other data used to guide content.	

Needs assessments with targeted learners	Data identified in Table 1		No evidence that a targeted needs assessment conducted.	

Goals and objectives	Course documents		Course goals not explicitly stated. Course objectives are not specific or measurable. Core syllabus inconsistent in formatting and presentation.	"Get defined objectives, we have...those for teaching and we have to use those for the evaluation."

Educational Strategies	Course documents, evaluation forms, interview data.	Complex and creative curriculum. Curriculum uses diverse educational strategies to deliver content maximizing opportunities for appropriate knowledge, skills and attitudes to be developed.	Clinical presentations not well developed.	"I had no idea that the course was organized into 25 presentations until this moment."

Implementation	Data identified in Table 1	Many people committed to delivering curriculum. Facilities can support the delivery of the curriculum. Many teaching strategies were used formally and informally to optimize learning. Administrative support was essential.	Small group learning sessions larger (n = 18) than would be optimal for small group learning. Active learning not optimized. Perception that course was disorganized. Time constraints (insufficient number of hours) prevented course from being delivered in an optimal way. An inability to provide consistent guidance and direction to the many teachers caused unnecessary duplication on content and an inconsistent understanding of the course's objectives and approach to teaching using a clinical presentation format.	"Too much information for the length of the course. Six weeks or six and a half weeks is not long enough."

Evaluation	Student examination data from MSK and courses preceding and following MSK. Evaluation forms from students and faculty. Interviews with key informants.	Examination delivered to students provides evidence that it was reliable. Student and faculty evaluation feedback data provides evidence that it is reliable. Data from the student and faculty feedback evaluation provided useful information to guide future revisions to curriculum. Course evaluations were reviewed and acted upon to call for a wider review and guide improvement.	Blueprint, objectives and student examination are not aligned. Lack of feedback to faculty about their performance as teachers.	"My ability to MEMORIZE was tested, not my ability to solve problems." "Would be great to get some feedback on the sessions that I taught." "Need to have lecturers submit 1 or 2 questions directly from each of their presentations for use as MCQs to improve question bank."

Integration of curricular components	Core documents. Interviews with key informants.		Poor alignment of objectives with content presented and with student examination.	"...Cover in lectures and in core document what will be tested on the exam."

**Table 4:** Perceptions of students and faculty members about the MSK Curriculum

What are the perceptions of students and faculty members about the strengths and weaknesses of the MSK course?			
**Data sources**	**Strengths**	**Weaknesses**	**Exemplar Quotes**

Course evaluation forms from students and faculty. Interviews with key informants.	Faculty were committed to the course. Course content was excellent particularly anatomy and its integration with clinical and physical examination skills	Too many students in small groups. Too much material for students to master. Material was not sequenced. Course objectives lacked clear direction. The large number of faculty made communication difficult. Core document could be improved.	"Independent study is a strength...[this] course that stands quite a significant volume of material ...It offers the student the opportunity for both didactic and directed learning and self directed learning or independent study" "[The] sequence isn't very logical. One minute you're talking about fractures, the next you're talking about burns ...I don't think often times a lot of attention is paid to is how well does it, kind of, flow."

**Table 5:** Evidence of validity and reliability of surveys and student MCQ examination

What is the evidence that the student and faculty feedback surveys used to evaluate the MSK course were valid and reliable?			
**Data sources**	**Strengths**	**Weaknesses**	**Exemplar Quotes**

Student and faculty evaluation feedback data.	Student feedback evaluation was very reliable (α >.92). Faculty feedback was reliable (α >.76). The data from the evaluations was aligned with the data produced through the interviews with key informants.		

**What is the evidence that the student examination was valid and reliable?**			

**Data sources**	**Strengths**	**Weaknesses**	**Exemplar Quotes**
Student examination data for MSK course and for courses preceding and following MSK.	Examination reliable (Cronbachs α r = 0.76). High correlation between students who scored well on MSK and earlier/later courses r > .638	MSK underrepresented on the exam relative to other content in course (special senses and dermatology). Topics on exam were not always aligned with content of course.	"Integrating ophthalmology and ENT into the MSK course before doing neuro made learning the subject matter very difficult."

There were weaknesses that the Kern framework[12]illuminated, including the apparent absence of a formalized needs assessment that had guided curriculum, course objectives that were not specific or measurable, clinical presentations (on which the curriculum was structured) that were not well developed, small group sessions that exceed suggested normal 'small group' sizes of 6-8 in curricular documents, and the poor alignment between the objectives, examination blueprint and the examination administered to students. Both students and faculty members perceived the same strengths and weaknesses in the curriculum (Table 4). The exams were reliable and there was evidence of examination validity based on the correlation of examination scores between the MSK course and those courses which preceded and followed this course(Table 5). Lastly, the course evaluation data provided information that was consistent with the findings from the interviews with the key stakeholders. The evaluation data provided information that could be used to guide teachers and administrators in improving the course.

## Discussion

This study was initiated as a result of consistently poor feedback from medical students taking the MSK course as part of the University of Calgary, Faculty of Medicine undergraduate curriculum. The study also coincided with curricular changes were also implemented the year prior (2006), and with the attention that the external accreditation processes bring in evaluating a medical school.

The study's core research question, "What were the strengths and weaknesses of the MSK course?" was approached using a detailed, triangulated process employing a mixed method approach. This approach identified a number of the strengths and weaknesses in the course, and confirmed the feedback provided by the students that the course was performing sub-optimally.

A case study approach provided a framework for the evaluation of the MSK course curriculum, its alignment with a structured approach to curricular design, and its approach to student assessment and course evaluation. All of these data were triangulated (cross referencing documentation, member checking, comparing data from different sources, using content experts for second opinions), confirming and adding to the credibility and trustworthiness of the findings. Case study research provides a flexible approach to assessing school curricula. It allows the researchers to draw on both qualitative and quantitative data, multiple documents and sources, interviews, and the views of key stakeholders. The study design allowed the research team to understand a complex social phenomena through understanding "real life" events, such as organizational process[9,11].

This study illustrates the challenges of curriculum redesign and management. Historical and political influences commonly guide curricular change[19] especially where change can be instituted without potentially considering all the elements and influences in the milieu of the curriculum. It is thus not surprising that continuous "renovation" of this curriculum demonstrated less than ideal evaluations by students and teaching faculty.

Only through a thorough and systematic approach to planning and evaluation can success to be achievable. Re-creating a curriculum is intimately linked to the needs of learners and society - needs which periodically require re-examination[12]. Such a change has been documented in surgical education where a paradigm shift has been described[19]. This shift has occurred as a response to work hour restrictions[20], financial constraints[21], patient safety endeavours[22] and availability and use of the Internet[19]. In response, surgical educators have changed their curriculum to include Internet-based learning tools and surgical skill simulators[19]. Thus learner needs and societal needs have influenced changes in how, or what, adult learn.

Bordage describes conceptual frameworks to guide work in medical education[23]. These conceptual frameworks are used to highlight key variables that should not be overlooked when approaching curricular design. The Kern framework[12] is one such conceptual framework to guide the curricular process, either from inception or re-evaluation[23]. With the Kern model, educators can follow steps to evaluate, change or leave curricular elements intact a priori. The model allows for a proactive approach rather than a reactive one. By following the model, educators can appreciate how the steps work together, with changes in one area affecting other areas in a dynamic process. This process is also highlighted with Constructive Alignment, as described by Biggs[24,25], which integrates the alignment of teaching with outcomes, to promote higher order learning.

The Kern framework has undergone revisions to accommodate for the changing environment of medical education in North America, such as accreditation, a focus on core competencies (ACGME, RCPSC) and the growing use of information technology by institutions and learners. As stated by it's authors "the general principles of curriculum development remain timeless" (page ix, 2nd Edition)[12]. This framework has a healthcare and medical education focus. It does involve a constant iterative and cyclical process which can appear never ending. As one step is modified, the other steps must be examined and modified as well. This iterative process is essential for curriculum design and evaluation if a curriculum is to retain its currency.

There are limitations to this study. It provides a description of a case study applied to one musculoskeletal course and curriculum within one university's undergraduate program. Nonetheless, the approach of using case study research and the Kern framework[12] provides a structured way that other schools can look at their curriculum in MSK or other disciplines.

This study demonstrated the importance of conducting an initial needs assessment and developing clear and measurable objectives prior to embarking on curricular change. Wadey et al[26] performed a thorough needs assessment of post graduate MSK course objectives in Canada and how they reflect the current curricular recommendations by the Bone and Joint Decade Undergraduate Curriculum Group (BJDUCG)[3]. Their results validated the 80 curricular objectives proposed by the BJDUCG and identified 10 other key objectives[26]. The revised Canadian MSK Core Curriculum was then created from the results[26]. The Canadian MSK Core Curriculum, along with the BJDUCG curriculum, provides credible and valid curricular assessments to guide MSK education in North America[26].

## Conclusion

This study illustrates that the case study approach for assessing a curriculum functioning at a suboptimal level, can be valuable. The case study approach allowed us to use multiple data sources; within a pre-determined structured framework; to look critically at all aspects of the curriculum. We were able to look at both curriculum strengths and weaknesses in conjunction with four guiding questions. The first question focused on whether the curriculum was structured drew upon the Kern six step model and showed us deficiencies and strengths[12]. The second question focused on student and teacher perceptions from focus groups and the evaluation forms which further elaborated on strengths and weaknesses from evaluation forms and our examination of course materials. The third and fourth questions addressed the reliability and validity of the examination and evaluation processes and gave us confidence that the tools were working and producing data that was valid and reliable.

## Competing interests

The authors declare that they have no competing interests.

## Authors' contributions

MLC carried out the study design, data collection, analysis, and writing of the manuscript. JML contributed to the study design, analysis and review of the manuscript and CRH contributed to the review and editing of the manuscript. All authors have reviewed and approved the final version of the manuscript.

## Authors' Information

Marcia Clark MD, MSc, Dip. Sport Med., FRCS(C)

Assistant Professor, Department of Surgery

Faculty of Medicine and Dentistry, University of Alberta

Room 425 Community Services Centre

10240 Kingsway Avenue

Edmonton, Alberta T5 H 3V9

Carol R Hutchison MD, MEd, FRCS(C)

Associate Professor, Department of Surgery

Faculty of Medicine, University of Calgary,

3330 Hospital Dr. NW,

Calgary, Alberta, T2N 4N1

Jocelyn M Lockyer, PhD

Associate Dean, Continuing Medical Education and Professional Development

Professor, Department of Community Health Sciences

Faculty of Medicine, University of Calgary

3330 Hospital Drive NW

Calgary AB Canada T2N 4N1

## Pre-publication history

The pre-publication history for this paper can be accessed here:

http://www.biomedcentral.com/1472-6920/10/93/prepub
